# Strong low-energy rattling modes enabled liquid-like ultralow thermal conductivity in a well-ordered solid

**DOI:** 10.1093/nsr/nwae216

**Published:** 2024-06-22

**Authors:** Peng-Fei Liu, Xiyang Li, Jingyu Li, Jianbo Zhu, Zhen Tong, Maiko Kofu, Masami Nirei, Juping Xu, Wen Yin, Fangwei Wang, Tianjiao Liang, Lin Xie, Yongsheng Zhang, David J Singh, Jie Ma, Hua Lin, Junrong Zhang, Jiaqing He, Bao-Tian Wang

**Affiliations:** Institute of High Energy Physics, Chinese Academy of Sciences, Beijing 100049, China; Spallation Neutron Source Science Center, Dongguan 523803, China; Beijing National Laboratory for Condensed Matter Physics, Institute of Physics, Chinese Academy of Sciences, Beijing 100080, China; Department of Physics & Astronomy and Stewart Blusson Quantum Matter Institute, University of British Columbia, Vancouver V6T 1Z4, Canada; Institute of High Energy Physics, Chinese Academy of Sciences, Beijing 100049, China; Spallation Neutron Source Science Center, Dongguan 523803, China; State Key Laboratory of Advanced Welding and Joining Harbin Institute of Technology, Harbin 150001, China; School of Advanced Energy, Sun Yat-Sen University, Shenzhen 518107, China; J-PARC Center, Japan Atomic Energy Agency, Tokai, Ibaraki 319-1195, Japan; J-PARC Center, Japan Atomic Energy Agency, Tokai, Ibaraki 319-1195, Japan; Institute of High Energy Physics, Chinese Academy of Sciences, Beijing 100049, China; Spallation Neutron Source Science Center, Dongguan 523803, China; Institute of High Energy Physics, Chinese Academy of Sciences, Beijing 100049, China; Spallation Neutron Source Science Center, Dongguan 523803, China; Spallation Neutron Source Science Center, Dongguan 523803, China; Beijing National Laboratory for Condensed Matter Physics, Institute of Physics, Chinese Academy of Sciences, Beijing 100080, China; Institute of High Energy Physics, Chinese Academy of Sciences, Beijing 100049, China; Spallation Neutron Source Science Center, Dongguan 523803, China; Shenzhen Key Laboratory of Thermoelectric Materials, Department of Physics, Southern University of Science and Technology, Shenzhen 518055, China; Guangdong Provincial Key Laboratory of Advanced Thermoelectric Materials and Device Physics, Southern University of Science and Technology, Shenzhen 518055, China; Advanced Research Institute of Multidisciplinary Sciences, Qufu Normal University, Qufu 273165, China; Department of Physics and Astronomy, University of Missouri, Columbia, 65211, USA; Key Laboratory of Artificial Structures and Quantum Control, School of Physics and Astronomy, Shanghai Jiao Tong University, Shanghai 200240, China; State Key Laboratory of Structural Chemistry, Fujian Institute of Research on the Structure of Matter, Chinese Academy of Sciences, Fuzhou 350002, China; Institute of High Energy Physics, Chinese Academy of Sciences, Beijing 100049, China; Spallation Neutron Source Science Center, Dongguan 523803, China; Shenzhen Key Laboratory of Thermoelectric Materials, Department of Physics, Southern University of Science and Technology, Shenzhen 518055, China; Guangdong Provincial Key Laboratory of Advanced Thermoelectric Materials and Device Physics, Southern University of Science and Technology, Shenzhen 518055, China; Institute of High Energy Physics, Chinese Academy of Sciences, Beijing 100049, China; Spallation Neutron Source Science Center, Dongguan 523803, China

**Keywords:** thermal conductivity, thermoelectric, phonon dynamic, inelastic neutron scattering

## Abstract

Crystalline solids exhibiting inherently low lattice thermal conductivity (*κ*_L_) are of great importance in applications such as thermoelectrics and thermal barrier coatings. However, *κ*_L_ cannot be arbitrarily low and is limited by the minimum thermal conductivity related to phonon dispersions. In this work, we report the liquid-like thermal transport in a well-ordered crystalline CsAg_5_Te_3_, which exhibits an extremely low *κ*_L_ value of ∼0.18 Wm^−1^K^−1^. On the basis of first-principles calculations and inelastic neutron scattering measurements, we find that there are lots of low-lying optical phonon modes at ∼3.1 meV hosting the avoided-crossing behavior with acoustic phonons. These strongly localized modes are accompanied by weakly bound rattling Ag atoms with thermally induced large amplitudes of vibrations. Using the two-channel model, we demonstrate that coupling of the particle-like phonon modes and the heat-carrying wave-like phonons is essential for understanding the low *κ*_L_, which is heavily deviated from the 1/*T* temperature dependence of the standard Peierls theory. In addition, our analysis indicates that the soft structural framework with liquid-like motions of the fluctuating Ag atoms is the underlying cause that leads to the suppression of the heat conduction in CsAg_5_Te_3_. These factors synergistically account for the ultralow *κ*_L_ value. Our results demonstrate that the liquid-like heat transfer could indeed exist in a well-ordered crystal.

## Teaser

Liquid-like heat transfer is observed and explained in a well-ordered crystal, which hosts soft structural framework with liquid-like motions of the fluctuating/rattling Ag atoms.

## INTRODUCTION

The exploration of solid materials with ultralow lattice thermal conductivity (*κ*_L_) is of great interest due to their important applications as thermal insulators [1], thermal barrier coatings [2] and thermoelectric materials [3,4]. Strategies for suppressing phonon propagation and reducing thermal conductivity include the introduction of multidimensional defects into the material matrix [5], atomic ordering [6], high-entropy engineering [7] and others [8]. However, many strategies require the property of low thermal conductivity. As a result, screening low *κ*_L_ materials has become an important step [9–11]. In insulators, the thermal conductivity can be controlled mainly by the lattice component, *κ*_L_, while, in doped semiconductors used as thermoelectrics, both the lattice and the electronic components contribute to thermal conductivity. Controlling the lattice portion is crucial for high thermoelectric performance, especially in low- and medium-temperature zones.

In crystalline materials, both the transverse and longitudinal acoustic phonons contribute to the *κ*_L_ [12,13]. In liquids, if we do not consider the convection, the thermal transport is mainly governed through longitudinal vibrations [14]. Therefore, solids are usually more conductive than non-convective liquids. Liquids can also exhibit a lower heat capacity than solids due to the absence of stable transverse modes, reducing their thermal conductivity. As a result, solids that have liquid-like vibrational spectra will exhibit low thermal conductivity, due to both reductions in specific heat and propagating transverse modes [15–17]. This has guided the design and experimental demonstration of ultralow-*κ*_L_ crystalline compounds [18], such as Cu_2_Se [19,20], AgCrSe_2_ [21], Cu_4_TiSe_4_ [22], Cu_7_PSe_6_ [23], Ag_8_SnSe_6_ [24,25] and Ag_9_GaSe_6_ [26]. Some of these crystals have liquid-like mobile ions, which significantly reduce the thermal conductivity. Generally, the so-called phonon-liquid electron-crystal materials have large unit cells, with highly disordered atoms and complex structures [8,10,27]. These materials commonly exhibit low *κ*_L_. Although many ultralow-*κ*_L_ crystalline compounds have been explored, the liquid-like *κ*_L_ in well-ordered crystals is rarely explored both in theoretical predictions and experiments.

CsAg_5_Te_3_ [28], a well-ordered single-phase material, was recently reported to achieve a high figure of merit (ZT) of about 1.5 at 727 K, without any extrinsic doping [29], making it a promising mid-temperature thermoelectric single-phase bulk material [30,31]. This is especially important considering that the optimization of the carrier concentration could yield an even higher ZT. The key feature of CsAg_5_Te_3_ was found to be its exceedingly low *κ*_L_ of ∼0.18 W m^−1^ K^−1^ at 300 K [29]. This value is even lower than those of the phonon-liquid electron–crystal materials [13,18] and is within a factor of only seven times the thermal conductivity of air (∼0.025 W m^−1^ K^−1^ at 300 K). Meanwhile, with increasing temperature, the *κ*_L_ of CsAg_5_Te_3_ shows nearly temperature-independent behavior, which is different from the expectations of a semiconductor with propagating phonons governed by the Peierls–Boltzmann equation [31–33].

In this work, we used inelastic neutron scattering (INS) and first-principles calculations, supplemented with transport measurements, to elucidate the relationship between the phonon picture of atomic vibrations and the extremely low-*κ*_L_ behavior of a well-ordered crystalline CsAg_5_Te_3_. Our INS experiments show that there exist strongly localized low-lying phonon bands at ∼3.1 meV. When combined with calculations, we found that they are mainly dominated by the rattling Ag atoms with the avoided-crossing feature of acoustic and optical phonon branches. These Ag atoms have liquid-like motions as melted sublattices in CsAg_5_Te_3_, which is connected with the ultralow *κ*_L_. Based on the two-channel thermal conductivity calculations, we corroborated the predominant role of the wave-like phonons, as well as the important role of the coupling between the coherent wave-like modes and the localized particle-like modes. Our study provided an overall understanding of the liquid-like heat transport in a well-ordered crystal, which would facilitate the designing of low-*κ*_L_ materials.

## RESULTS

### Experimental and theoretical phonons

CsAg_5_Te_3_ crystallizes in the tetragonal space group, *P*4_2_/*mnm*, and its Zintl-type structure has two open tunnels and two infinite parallel [Ag_5_Te_3_]^−1^ chains along the *c*-axis [29] (see Fig. S1). In the chains, the Ag atoms are tetrahedrally or triangularly coordinated with the Te atoms to form a structural framework with weak chemical bonds [34]. The large-radius Cs^+^ ions (1.74 Å) have a large atomic mass fill in the tunnels at the center (0.5, 0.5, 0.5) and the origin (0, 0, 0) of the unit cell, stabilizing the structure. The pure polycrystalline sample is verified by the neutron diffraction data at 300 K, which are analysed by using the method of Rietveld refinement with the two strongest peaks of (550) and (552) marked in Fig. S2.

**Figure 1.**
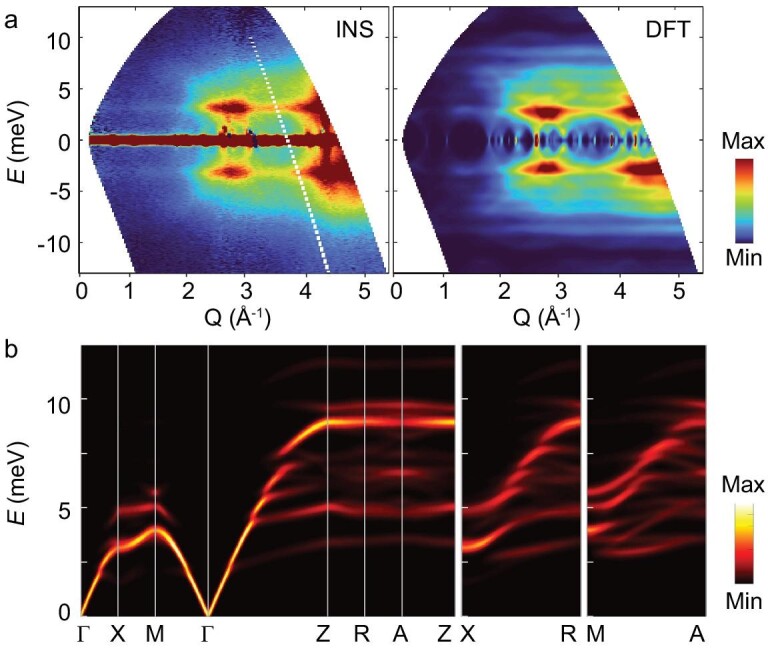
Experimental and calculated phonons of CsAg_5_Te_3_. (a) The contour plots of the dynamic structure factor *S*(**Q**, E) obtained by INS with an incident neutron energy of *E*_i_ = 15.15 meV at 295 K from the AMATERAS measurements (left) and the corresponding powder-averaged coherent *S*(**Q**, E) at 300 K generated by the temperature-dependent force constants from MD via first-principles calculations (right). These results clearly show that there were low-lying phonon modes at ∼3.1 meV. (b) Corresponding *S*(**Q**, E) weighted phonon dispersion relationship in the first Brillouin zone at 300 K calculated by Euphonic. To facilitate comparison between experiments and theory, we have normalized *S*(**Q**, E) in these figures.

We first plot in Fig. 1a and Fig. S3 the dynamic structure factor S(**Q**, E) at different temperatures with INS on the powder samples to analyse the lattice dynamics. As shown on the left side of Fig. 1a, our INS measurements of the orientation averaged S(**Q**, E) at 295 K reveal a striking mushroom-like scattering pattern, near the quasi-Brillouin-zone center at **Q** = 2.7 Å^−1^ and around a phonon frequency of 3.1 meV. The computed S(**Q**, E) at 300 K based on first-principles methods is plotted on the right side of Fig. 1a, which shows excellent agreement with the INS data in both phonon energies and intensities. To rationally understand these mushroom-like low-energy modes, we calculate the S(**Q**, E)-weighted phonon dispersion in the first Brillouin zone along the high-symmetry lines in Fig. 1b and Fig. S4 at 300 K using the same parameters as shown in Fig. 1a. This could serve as a powerful method to probe the single-crystal S(**Q**, E) based on the polycrystalline samples. As shown in Fig. 1b and Fig. S4, only the longitudinal vibrations of the acoustic phonons propagate along Γ–X–M–Γ–Z and are cut off starting at ∼2 meV by numerous optical branches and typically accompanied by the rattling modes [35]. One low-energy optical mode is observed along X–M. In the case of the other directions, there are only a few optical modes evident at <10 meV, with one flat mode at 15 meV along Z–R–A–Z. More specifically, most modes do not appear visibly in the spectrum while the longitudinal acoustic vibrations survive. Usually, phonon dispersions should be measured by INS using different regions of reciprocal space for a single crystal [36]. The simulations only in the first Brillouin zone cannot capture the whole picture of the phonons. Since the high-resolution neutron diffraction data show that there are two strong peaks at (550) and (552) (see Fig. S2), we calculate the S(**Q**, E)-weighted spectra for the Brillouin zones centered at the (550) and (552) zones and present them in Fig. S5. It clearly shows that all the acoustic phonons participate in the propagation. This is different from the superionic conductors [20], in which the structurally dynamic disorder damages the transverse acoustic phonons. Most notably, the powder INS data presented here provide an average density of states for all directions and regions of reciprocal space, while Fig. 1b and Fig. S2 only correspond to the two zones along specific directions. Beyond that, our theoretical and experimental results indeed demonstrate the existence of low-lying phonon modes. From first-principles calculations, we see that there exist the avoided crossings of optical–acoustic branches with the emerging concerted rattling modes [29] in Fig. S6. These in turn modulate the group velocities and scattering process, and also suppress the *κ*_L_. In addition, we have calculated the participation ratio (PR) and spatial distribution of the phonon modes in Fig. S7. Clearly, the PR values in Fig. S7 of the low-lying optical modes at ∼3.1 meV are close to ∼0.2, which means that these modes are likely localized [37]. The spatial distribution shows that the localized phonons with energy of <4 meV are mostly in the Ag atoms. The existence of abundant localized phonons often occurs in non-crystalline materials, quasicrystals and nanostructured materials, which would cause a transition from propagative to diffusive-like energy transport [37,38]. For our system, it brings about the abnormal ultralow two-channel heat transport mechanism in CsAg_5_Te_3_, as discussed below.

**Figure 2.**
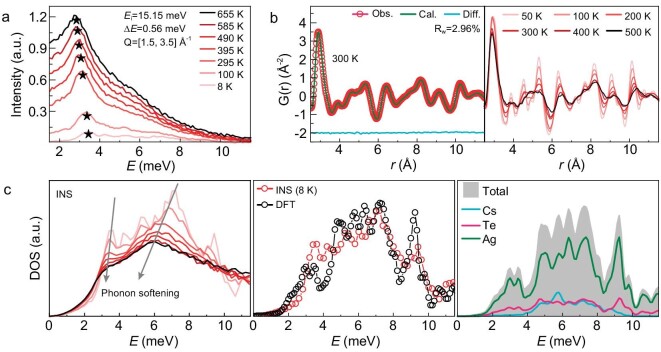
Temperature-dependent vibrational properties and local structures of CsAg_5_Te_3_. (a) The cuts of *S*(**Q**, E) at *E*_i_ = 15.15 meV integrated over the range of 1.5 ≤ **Q** ≤ 3.5 Å^−1^ under all measured temperatures. (b) Neutron PDF data at the indicated temperatures, where the room-temperature neutron PDF data are refined using the *P*4_2_/*mnm* space group (ambient crystal structure) with all atom positions and thermal parameters refined. (c) (Left) Neutron-weighted phonon DOS from the AMATERAS measurements at the indicated temperatures with phonon softening. The DOS at lower phonon frequencies (<1 meV) are fitted to the Debye model. (Middle) Calculated generalized phonon DOS from DFT lattice dynamics and neutron-weighted phonon DOS from INS at 8 K. (Right) The partial phonon DOSs on Cs, Ag and Te from DFT.

The temperature-dependent behavior of the low-energy phonons is analysed by integrating the S(**Q**, E) data over 1.5 ≤ **Q** ≤ 3.5 Å^−1^, as shown in Fig. 2a. At 8 and 100 K, we observe one prominent peak, as marked by the stars, as well as several less intense peaks. Upon heating from 295 to 655 K, there is only one intense peak in the indicated region. This big peak is far away from the intense elastic line and corresponds to the low-lying phonon modes at around **Q** = 2.7 Å^−1^, as shown in Fig. 1 and Fig. S4. The scattering is further analysed in real space by Fourier-transforming the static structure factor into the pair distribution functions (PDFs). Figure 2b shows the PDFs for the pair distances from 2.5 to 11.5 Å at selected temperatures. Figs S8 and S9 show the first peak located at ∼2.83 Å. The superposition of the nearest neighboring Ag–Te and Ag–Ag bonds, which correspond to the structural tunnels in the crystal structure without local structural distortions, induces the absence of the shoulder peak at ∼2.83 Å [39].

When the temperature is increased, the amplitude of the first peak in Fig. 2b is monotonically decreased, while other peaks of >3.5 Å are significantly affected and smoothed out. This clearly indicates that the structural framework is preserved well upon heating but exhibits substantial atomic motions. The long-distance pairs gradually lose their correlation upon heating. This fact is further confirmed by our simulated trajectories of the atoms in the *x*–*y* plane at 300, 500 and 700 K by the molecular dynamics (MD) simulations, as presented in Figs S10 and S11. In Fig. S12, the self-part of the van Hove correlation function G_s_(*r, t*) calculated from the MD simulations depicts the probability of atoms diffusing away from the initial positions by a distance *r* after a period *t*. As time goes on, the G_s_(*r, t*) fluctuate at a fixed value for all atoms. There are no jump diffusions for all atoms in CsAg_5_Te_3_. Meanwhile, our calculated Lindemann parameter, δ = *ADP*^1/2^/*R*_NN_ (where *R*_NN_ is the nearest neighbor distance and *ADP* is the atom displacement parameters), is 0.0415, 0.0494/0.050 and 0.061/0.076/0.079 for Cs, Te and Ag atoms, respectively. Clearly, the values of Ag atoms exceed or approach to the criterion of melting (≥0.07) as in liquids [40]. This indicates that the oscillation amplitudes of the Ag atoms are much larger than those of the Cs/Te atoms and they have liquid-like motions as a melting sublattice in the crystal with weak chemical binding. Nonetheless, the complete structural skeleton is preserved with the coexisting crystal–liquid duality [41], unlike materials with liquid-like states characterized by atomic diffusions [40].

To investigate the temperature effect on phonons, we resolve the phonon density of states (PhDOSs) from the temperature-dependent INS measurements. As shown in Fig. 2c, the PhDOS at 8 K presents several pronounced peaks at 3.5, 4.2, 5.4, 6.9 and 9.1 meV. These positions are related to the flat branches in Fig. S4. Consistently with S(**Q**, E) in Fig. 2a, upon heating, the peak in Fig. 2c at ∼3.5 meV is broadened and softened, while the other features eventually disappear into a broad bulge. Meanwhile, the density functional theory (DFT)-based simulations yield a total PhDOS with low-energy peaks at 3.0, 3.5, 4.9, 5.8, 6.4, 7.3 and 9.3 meV. Although there are differences in the intensity, number and position of phonons obtained from theory and experiment, the overall trend from theory is very close to that of the INS PhDOS at 8 K. In fact, we should be aware that theoretical calculations depend on the pseudopotential and some settings, while there must be errors between experimental measurements and true values. All these factors can lead to differences between theoretical and experimental values. The partial PhDOSs can be given by:


\begin{eqnarray*}
{{g}_i}( \omega) = \mathop \sum \limits_{j,{\boldsymbol{q}}} {{\left| {{{e}_i}\left( {j,{\boldsymbol{q}}} \right)} \right|}^2}\delta \left( {\omega - \omega \left( {j,{\boldsymbol{q}}} \right)} \right),
\end{eqnarray*}

where *ω* and *e*_i_ denote the phonon energies and eigenvectors, respectively, and *i* is the atom index. The partial PhDOSs clearly show that the low-energy peaks ranging from 2 to 4 meV are overwhelmingly contributed by the structural tunnels dominated by the soft Ag–Te bonds [34]. The existence of soft bonds is also evaluated by elastic properties in Table S1. Importantly, this soft bonding is correlated with the strong lattice anharmonicity measured by the phonon softening with increasing temperature, as indicated in Fig. 2c.

### Two-channel thermal conductivity

**Figure 3.**
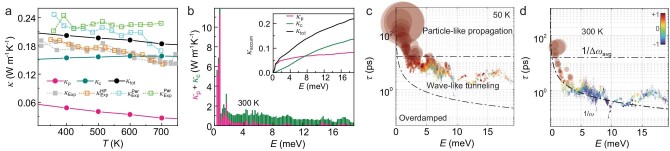
Thermal conductivity of CsAg_5_Te_3_. (a) Measured temperature-dependent lattice thermal conductivity ($\kappa _{{\mathrm{Exp}}}^{{\mathrm{HP}},\!{\mathrm{\ Per}},\!{\mathrm{\ Par}}}$) and the calculated values (*κ*_tot_ = *κ*_p_ + *κ*_c_) from first-principles simulations, where $\kappa _{{\mathrm{Exp}}}^{{\mathrm{Per}},{\mathrm{Par}}}$ indicate the values parallel and perpendicular to the pressing directions with the SPS method, $\kappa _{{\mathrm{Exp}}}^{{\mathrm{HP}}}$ represents the values of the hot-pressed sample, ${{\kappa }_{{\mathrm{Exp}}}}$ are values extracted from our previous work [29], *κ*_p_ and *κ*_c_ account for the heat transfer associated with the diagonal (populations) and the off-diagonal (coherences) Wigner distribution elements, respectively, and *κ*_tot_ is the total lattice conductivity. The results of $\kappa _{{\mathrm{Exp}}}^{{\mathrm{Per}}}$ and $\kappa _{{\mathrm{Exp}}}^{{\mathrm{Par}}}$ are nearly the same, indicating the good reliability of our results. (b) Phonon-mode-resolved thermal conductivities of populations (*κ*_p_) and coherences (*κ*_c_) at 300 K. Inset: the cumulative total thermal conductivity (*κ*_tot_) as a sum of the population contribution (*κ*_p_) and coherences contribution (*κ*_c_) at 300 K. (c and d) Phonon lifetimes τ(**q**) = [Γ(**q**)]^−1^ as a function of the energy *ω*(**q**) at 50 (left) and 300 K (right), respectively, where the area of each circle is proportional to the contribution to the *κ*_L_ and the colors indicate the origin of the contribution by $c = \frac{{{{\kappa }_{\mathrm{p}}} - {{\kappa }_{\mathrm{c}}}}}{{{{\kappa }_{\mathrm{p}}} + {{\kappa }_{\mathrm{c}}}}}$: *c* = 1 for particle-like propagation of the populations, *c* = −1 for wave-like tunneling of coherences and the intermediate values for phonons contributed from both mechanisms.

As discussed above, CsAg_5_Te_3_ has a well-ordered crystalline atomic structure, but features the liquid-like motions of Ag atoms. To accurately predict the *κ*_L_, we solve the Wigner transport equation by simultaneously considering the particle-like and the wave-like conduction mechanisms from both the population and coherence contributions [41,42]. In our transport experiment, the measured *κ*_L_ value is ∼0.19–0.25 W m^−1^ K^−1^, based on subtracting *κ*_e_ in Fig. S13 from the total *κ* in Fig. S14. To compare experimental data with theory, the calculated *κ* of the sample is averaged along the three crystallographic axes. The calculated anisotropic *κ* is shown in Fig. S15. As shown in Fig. 3a, the calculated population contribution *κ*_p_ is equal to 0.09 W m^−1^ K^−1^ at 300 K, which is much lower than our measured values and the reported result (0.18 W m^−1^ K^−1^) [29]. This means that, for CsAg_5_Te_3_, the Peierls picture breaks down in predicting *κ*_L_ when considering only the propagating vibrational waves as heat carriers. Thus, the coherence lattice thermal conductivity *κ*_c_, from the wave-like interband (Zener) tunneling of phonons, is also considered and is calculated as 0.14 W m^−1^ K^−1^ at 300 K (see Fig. 3a). The convergent *κ*_L_ value in Fig. S16, as a sum of both *κ*_p_ and *κ*_c_, is ∼0.23 W m^−1^ K^−1^ at 300 K, which coincides well with the experimental results. With increasing temperature, the *κ*_p_ decreases inversely with temperature, following the Peierls theory. This is in contrast to the observed temperature dependence of the *κ*_L_. The contribution of the *κ*_c_ is almost unchanged and is dominant in the range of the measured temperatures. This contribution offsets the incorrect Peierls–Boltzmann conductivity and leads to our prediction of *κ*_L_ (*κ*_L_ = *κ*_p_ + *κ*_c_) in good agreement with the experimental values. This is also consistent with the hypothesis of the well-ordered crystallized CsAg_5_Te_3_ containing ultralow *κ*_L_ with liquid-like vibrational properties [43,44]. Figure 3b shows the contributions of *κ*_p_ (pink) and *κ*_c_ (olive), as well as the cumulative conductivity at 300 K. The diagonal population contribution *κ*_p_ mainly comes from the low-lying modes of the overall crystal framework, as these modes having the largest group velocities, while all phonons that contribute to the coherence term are in a random distribution. To analyse the strength of the particle-like and the wave-like conduction, we classify the phonon lifetime *τ*(**q**)_s_ into three regimes in Fig. 3c by using the Ioffe–Regel limit (1/*ω*) and the Wigner limit (1/∆*ω*_avg_) [41,42]. Here, the average phonon interband spacing is calculated by using ∆*ω*_avg_ = *ω*_max_/3*N*_a_, where *ω*_max_ is the maximum phonon frequency and 3*N*_a_ is the number of phonon bands. These regimes operate under different mechanisms. At 50 K, as indicated in Fig. 3c, most phonons are >1/∆*ω*_avg_ and the particle-like phonons (red) propagate 87.4% of the total *κ*_L_. In medium- and high-temperature regions, for example at 300 K as shown in Fig. 3d, the largest number of phonons are in the intermediate region between the Wigner limit and the Ioffe–Regel limit, where the heat mainly diffuses in a wave-like fashion (*κ*_p_ = 0.09 W m^−1^ K^−1^; *κ*_c_ = 0.14 W m^−1^ K^−1^) with the major contributors to *κ*_c_ located near the diagonal of the frequency plane between quasi-degenerate vibrational frequencies shown in Fig. S17. Note that, within the temperature range of 50–700 K, as indicated in Fig. 3 and Fig. S18, almost all phonons are above the Ioffe–Regel limit and still exhibit the well-defined quasiparticle excitations [45], despite containing the liquid-like features [43,44]. Typically, carriers exhibit wave-like behavior and diffuse via Zener-like tunneling between such quasi-degenerate vibrational eigenstates in non-crystal-like materials such as amorphous solids, glasses or liquids. Thus, the quasiparticle excitations in CsAg_5_Te_3_, which manifest liquid-like features, give rise to the abnormal ultralow two-channel thermal conductivity.

## DISCUSSION

Generally, a nonmetallic solid will always have a higher *κ*_L_ when compared with a non-convective liquid. However, the Ag and Te atoms in CsAg_5_Te_3_ have soft bonds [34]. As a result, the structural [Ag_5_Te_3_]^−1^ tunnels undergo strong motion upon heating and the Ag atoms are almost melted in the crystal. Simultaneously, the isolated Cs^+^ cations, accommodated in the tunnels, stabilize the structure and largely suppress the disorder of the Ag atoms [46]. This is very different when compared with other materials involving structural-disorder-driven liquid-like features, such as Cu_2_Se [15], AgCrSe_2_ [21] and argyrodites [25]. In these solids, the site disorder of the ions allows thermally induced ionic diffusion, which is connected to their liquid-like behavior and the reduced *κ*_L_. In the well-ordered crystal of CsAg_5_Te_3_, although the Ag atoms exhibit large vibrational amplitudes and liquid-like motions like melting, they are still constrained and remain around their equilibrium positions due to the intrinsic structure. Instead, the special structural tunnels with soft bonds strongly scatter the phonons. The low-energy phonons range from 2 to 4 meV, are mainly contributed by the vibrations of such Ag–Te structured tunnels, exhibit large values of *γ*_qν_ (as shown in Fig. S19) and contribute to ∼70% of the total *κ*_p_ (see Fig. 3b). Meanwhile, this structure also leads to numerous low-lying optical phonons that cut the longitudinal acoustic mode, starting at ∼2 meV in Fig. S4, which can be associated with the presence of the concerted rattling modes [35], as described in our previous work [29]. These traits, together with strong lattice anharmonicity (*γ*_tot_ = 1.52 in Fig. S19), effectively disrupt the heat propagation, which accordingly gives rise to the extremely low two-channel *κ*_L_ and almost temperature independence of the *κ*_L_ from 300 to 700 K in CsAg_5_Te_3_. The liquid-like features in this well-ordered crystal indicate a promising general strategy for obtaining high-performance energy conversion materials with ultralow *κ*_L_. Structurally, materials with rattling-atom-based tunnels (such as Cu, Ag atoms) occupied by heavy atoms as well as having a well-ordered structure and hierarchical soft bonds [34,35,46–50] could be used to achieve ultralow thermal conduction. Differently from the general phonon–glass electron-crystal strategy that disrupts phonon transport by introducing disorder through alloying, nanostructuring and defect, our studies provide a useful way for realizing ultralow *κ* in materials with a low-energy strong scattering pattern from rattling modes as a signature.

## CONCLUSIONS

In this study, we found that crystalline CsAg_5_Te_3_, despite lacking disorder, has a liquid-like ultralow *κ*_L_ value of ∼0.18 W m^−1^ K^−1^ in the temperature range of 300–700 K. Based on experimental and theoretical evidence, we verified that there are abundant low-lying phonon modes propagated at ∼3.1 meV dominated by the liquid-like motions of the Ag atoms. In the two-channel model, the coherence contributions toward the *κ*_L_ come from wave-like phonon tunneling, dominated by the process of heat transport. By solving the Wigner formulation of thermal transport, our calculated *κ*_L_ offers substantially good agreement with the measured data. Our results attest to the liquid-like heat transfer in a well-ordered crystal as a paradigm-shifting approach beyond the classic ‘phonon–glass electron-crystal’ paradigm in the design of low-*κ*_L_ materials. We believe that our work can significantly promote the development of thermoelectrics, thermal management, thermal barrier coatings, thermal insulators, materials science and energy conservation.

## MATERIALS AND METHODS

### Synthesis

All synthesizing manipulations were conducted in a glove box (moisture and oxygen levels of <0.1 ppm) or under a vacuum. Polycrystalline CsAg_5_Te_3_ samples were prepared from a mixture of Ag (shot, 99.999%, Alfa Aesar), Te (shot, 99.9999%, Alfa Aesar) and Cs_2_Te_3_ at a stoichiometric ratio of 10 : 3 : 1. The reactants were loaded into a fused-silica tube under a vacuum and heated to 1073 K, maintained at this temperature for 2 h and then cooled to room temperature. The obtained ingot was ground into fine powder using agate mortar to reduce the grains to <4 mm in diameter. The powdered compounds were obtained as pure phases.

### Thermal conductivity

The obtained powders were then placed inside a 12.7-mm-diameter graphite die and densified by using spark plasma sintering (SPS, SPS-211LX, Fuji Electronic Industrial Co., Ltd.) at 723 K for 10 min under an axial compressive stress of 50 MPa in a vacuum. After this treatment, we obtained highly dense disk-shaped pellets, with densities of >97% of the theoretical value (7.117 g/cm^3^). The pellets were 12.7 mm in diameter and 12 mm thick. Some of the obtained products were ground into fine powders that were subsequently condensed to a high density of >97% using a hot-pressed-only process at 500 K under a pressure of 60 MPa for 1 h (denoted as the hot-pressed sample). The electrical resistivity was measured in a low-pressure helium atmosphere using a ULVAC-RIKO ZEM-3 instrument system. The thermal diffusivity (*D*) was measured on a sample disk with a diameter of 12.7 mm and a thickness of 1.0 mm under an argon atmosphere in the range of 296–773 K by the laser flash diffusivity method using a commercial Netzsch LFA457 instrument. The heat capacity (*C*_p_) was obtained by using a Netzsch DTA 404 PC instrument. Prior to testing, a uniform graphite layer needed to be sprayed onto the surface of the material to achieve thermal conductivity. During the testing process, the Cape Lehman+ pulse model was selected for correction. The total thermal conductivity was calculated by using the following formula:


\begin{eqnarray*}
\kappa = {{D}} \times {{{{C}}}_{\mathrm{p}}} \times {{d}},
\end{eqnarray*}

where *d* is the sample density, determined by using the dimensions and mass of the sample and then reconfirmed by measurements using a gas pycnometer (Micromeritics AccuPyc 1340). According to *κ*_e_ = LσT = *κ* − *κ*_L_, the lattice thermal conductivity *κ*_L_ could be obtained by subtracting *κ*_e_ from *κ* with a Lorenz number (L) of 1.5 × 10^−8^ V^2^ K^−2^.

### Neutron diffraction measurements

Neutron powder diffraction measurements were performed using a multi-physics instrument [51] at the China Spallation Neutron Source (CSNS) [52], in China. The data were collected at 200, 300, 400 and 500 K for the CsAg_5_Te_3_ sample. The Rietveld refinements of the neutron diffraction data at 300 K for the crystal structures of the compound were conducted using the general structure analysis system (GSAS-II) package. The real-space refinement of the experimental *G*(*r*) was performed by using the PDFgui [53] program. In the refinement, the positions of all atoms in the unit cell were written and refined, and the symmetry constraints were generated by the symmetry of the space group.

### INS measurements

The INS measurements were performed by using a cold neutron disc chopper spectrometer BL14 AMATERAS [54] with a beam power of ∼830 kW at the Materials and Life Science Experimental Facility (MLF), J-PARC in Japan. The chopper configurations were set with incident energies *E*_i_ of 15.15 and 41.98 meV, and corresponding energy resolutions of 0.56 and 2.44 meV (full width at the half maximum of the elastic peaks). A 5.97-g CsAg_5_Te_3_ powder sample was encased in a double cylindrical aluminum cell (14 mm in diameter, 1 mm thick) and the neutron beam size was ∼20 mm wide and ∼40 mm high. Thus, the sample was completely immersed in the neutron beam. A top-loading closed cycle refrigerator (TL-CCR) was used for the temperature-dependent measurements at 8, 100, 295, 395, 490, 585 and 655 K. The average data collection time at high temperatures was ∼3 h, which was doubled at 295 K. Data reduction was completed using the Utsusemi software suite [55]. The background, contributed by the TL-CCR with the same double cylindrical aluminum cell, was measured at the same temperatures with the same instrument configurations and was subtracted properly. The resulting dynamic structure factor S(**Q**, E) was defined as a function of the neutron energy transfer *E* = *E*_i_ − *E*_f_ and the momentum transfer ***Q*** = **k**_i_ − **k**_f_ = **q** + **τ**, where *E*_f_ is the scattered neutron energy, **k**_i_(**k**_f_) denotes the incident (scattered) neutron wave-vector, **q** is the phonon wave-vector and **τ** is the reciprocal lattice vector. *S*(**Q**, E) was further visualized in the Mslice of the data analysis and visualization environment (DAVE) [56]. Neutron absorption correction was conducted by considering both the coherent and incoherent scattering cross sections and the absorption cross section was corrected by utilizing the Mslice/DAVE. The generalized ***Q***-dependent phonon density of states (GPDOSs), *G*(**Q**, E), were related to the dynamic structure factor, *S*(**Q**, E), by the following equation [57]:


\begin{eqnarray*}
G\left( {{\boldsymbol{Q}},{\boldsymbol{\ }}E} \right) = {{e}^{{{Q}^2}{{u}^2}}}\left[ {1 - {{e}^{ - \frac{E}{{{{k}_B}T}}}}} \right]\frac{E}{{{{Q}^2}}}S\left( {{\boldsymbol{Q}},{\boldsymbol{\ }}E} \right),
\end{eqnarray*}

where $[ {1 - {{e}^{ - \frac{E}{{{{k}_B}T}}}}} ]$ indicates the Bose–Einstein statistics, ${{e}^{{{Q}^2}{{u}^2}}}$ describes the Debye–Waller factor, *u* is the atomic thermal displacement, *k*_B_ is the Boltzmann constant and *T* is the temperature. The Debye–Waller factor was ignored by setting *u* = 0 for the ***Q***-integrated GPDOS calculation. The impact was minimal, as we integrating a short ***Q*** range of 1.5–3.5 Å^−1^ for *E*_i_ = 15.15 meV and 3.0–5.5 Å^−1^ for *E*_i_ = 41.98 meV, with the data shown in arbitrary units.

### Computational details

First-principles calculations were performed within the framework of the Perdew–Burke–Ernzerhof [58] generalized gradient approximation (PBE–GGA) [59,60], as implemented in the Vienna *ab initio* simulation package (VASP) [61]. The cut-off energy for the plane-wave expansion was set as 500 eV on a 3 × 3 × 11 Γ-centered **k**-mesh. All structures were fully relaxed until the residual forces on each atom were <0.01 eV/Å and the high-symmetry lines of the tetragonal lattices were used according to previous calculations [62]. The second-order force constants were calculated within the harmonic approximation using the finite-displacement method [63] on a 5 × 5 × 5 **k**-mesh for CsAg_5_Te_3_, with a 1 × 1 × 3 supercell (containing 108 atoms) using Phonopy code [64] bundled with VASP. The MD calculations were performed with a 1 × 1 × 3 supercell via a canonical ensemble and a Nosè–Hoover thermostat. At temperatures of 200, 300, 400, 500, 600, 700 and 800 K, the MD simulations were calculated using a plane-wave cut-off of 500 eV and a total time of 50 ps, setting 1 fs as the time step. The corrections to the second-order force constants due to the finite-temperature anharmonic effects were applied using the DynaPhoPy code [65] from MD at different temperatures.

The third-order force constants were computed on a 3 × 3 × 3 **k**-mesh and interactions up to the third-nearest neighbors were considered, using the Phono3py [66] package. The temperature-dependent second-order force constants and third-order force constants were Fourier-interpolated on a convergent (8 × 8 × 8) grid for thermal conductivity calculations and then generalized to an expression including both the population and coherence contributions [41,42], ${{\kappa }_L} = {{\kappa }_p} + {{\kappa }_c}$, with ${{\kappa }_p} = \frac{1}{{{{{( {2\pi } )}}^3}}}\mathop \smallint \nolimits_\mathcal{B}^{} \sum_s C{{( {\boldsymbol{q}} )}_s}{{V}^\alpha }{{( {\boldsymbol{q}} )}_{s,s}}{{V}^\beta }{{( {\boldsymbol{q}} )}_{s,s}}\frac{1}{{{\mathrm{\Gamma }}{{{( {\boldsymbol{q}} )}}_s}}}{{d}^3}q$ and ${{\kappa }_c} = \frac{1}{{{{{( {2\pi } )}}^3}}}\mathop \smallint \nolimits_\mathcal{B}^{} \sum_{s \ne s^{\prime}} \frac{{\omega {{{( {\boldsymbol{q}} )}}_s} + \omega {{{( {\boldsymbol{q}} )}}_{s^{\prime}}}}}{4}[ {\frac{{C{{{( {\boldsymbol{q}} )}}_s}}}{{{\mathrm{\omega }}{{{( {\boldsymbol{q}} )}}_s}}} + \frac{{C{{{( {\boldsymbol{q}} )}}_{s^{\prime}}}}}{{{\mathrm{\omega }}{{{( {\boldsymbol{q}} )}}_{s^{\prime}}}}}} ]{{V}^\alpha } {{( q )}_{s,s^{\prime}}}{{V}^\beta }{{( q )}_{s^{\prime},s}} \times \frac{{\frac{1}{2}[ {{\mathrm{\Gamma }}{{{( {\boldsymbol{q}} )}}_s} + {\mathrm{\Gamma }}{{{( {\boldsymbol{q}} )}}_{s^{\prime}}}} ]}}{{{{{[ {{\mathrm{\omega }}{{{( {\boldsymbol{q}} )}}_s} - {\mathrm{\omega }}{{{( {\boldsymbol{q}} )}}_{s^{\prime}}}} ]}}^2} + \frac{1}{4}{{{[ {{\mathrm{\Gamma }}{{{( {\boldsymbol{q}} )}}_s} + {\mathrm{\Gamma }}{{{( {\boldsymbol{q}} )}}_{s^{\prime}}}} ]}}^2}}}{{d}^3}q,$ where *κ*_p_ is the standard Peierls contribution to conductivity and the additional tensor, *κ*_c_, is derived from the coherence equation. The *κ* of the polycrystalline sample was further averaged along the three principal crystallographic axes [67]. Besides, we also calculated the *κ*_L_, *κ*_p_ and *κ*_c_ at 300 K for CsAg_5_Te_3_ with force constants being extracted by using temperature dependent effective potential technique [68,69] as contrasts in Table S2.

To compare with the experimental data from the multi-*E*_i_ time-of-flight INS, the GPDOS of CsAg_5_Te_3_ was calculated by summing the partial PhDOS values weighted by the atomic scattering cross sections and masses:


\begin{eqnarray*}
{\mathrm{GPDOS}} = \mathop \sum \limits_i \frac{{{{\sigma }_i}}}{{{{\mu }_i}}}{\mathrm{PhDO}}{{{\mathrm{S}}}_i},
\end{eqnarray*}

where *σ*_i_ and *PhDOS*_i_ represent the atomic scattering cross section and the PhDOS projected into the individual atoms, respectively. The two-dimensional *S*(**Q**, E) patterns, as shown in Fig. 1, were calculated from the second-order force constants with the Euphonic package [70]:


\begin{eqnarray*}
S\left( {{{\bf Q}},{\mathrm{E}}} \right) &=& \frac{1}{2}\mathop \sum \limits_\nu {{\left| {F\left( {{{\bf Q}},\nu } \right)} \right|}^2}\\
&&\times \left( {{{n}_{{\boldsymbol{q}}\nu }} + \frac{1}{2} \pm \frac{1}{2}} \right)\delta \left( {\omega - \mp {{\omega }_{{\boldsymbol{q}}\nu }}} \right),
\end{eqnarray*}

where the upper and lower signs refer to the phonon creation and annihilation, respectively, *n***_q_***_ν_* is the Bose population function and *F*(**Q**,*ν*) is the coherent one-phonon scattering structure factor.

## Supplementary Material

nwae216_Supplemental_File
