# Supplementary material for: Strong low-energy rattling modes enabled liquid-like ultralow thermal conductivity in a well-ordered solid
Source: Natl Sci Rev. 2024 Jun 22;11(12):nwae216. doi: 10.1093/nsr/nwae216 (PMC11562843; doi:10.1093/nsr/nwae216)
Supplement: nwae216_Supplemental_File [file nwae216_supplemental_file.docx]

Supplementary Materials for

**Strong low-energy rattling modes enabled liquid-like ultralow thermal conductivity in a well-ordered solid**

Peng-Fei Liu^1,2,†^, Xiyang Li^3,4,†^, Jingyu Li^1,2^, Jianbo Zhu^5^, Zhen Tong^6^, Maiko Kofu^7^, Masami Nirei^7^, Juping Xu^1,2^, Wen Yin^1,2^, Fangwei Wang^2,3^, Tianjiao Liang^1,2^, Lin Xie^8,9^, Yongsheng Zhang^10^, David J. Singh^11^, Jie Ma^12^, Hua Lin^13,*^, Junrong Zhang^1,2,*^, Jiaqing He^8,9,*^, and Bao-Tian Wang^1,2,*^,

^1^Institute of High Energy Physics, Chinese Academy of Sciences, Beijing 100049, China.

^2^Spallation Neutron Source Science Center, Dongguan 523803, China.

^3^Beijing National Laboratory for Condensed Matter Physics, Institute of Physics, Chinese Academy of Sciences, Beijing 100080, China.

^4^Department of Physics & Astronomy and Stewart Blusson Quantum Matter Institute, University of British Columbia, Vancouver, Canada V6T 1Z4.

^5^State Key Laboratory of Advanced Welding and Joining Harbin Institute of Technology, Harbin 150001, China.

^6^School of Advanced Energy, Sun Yat-Sen University, Shenzhen 518107, China.

^7^J-PARC Center, Japan Atomic Energy Agency, Tokai, Ibaraki 319-1195, Japan.

^8^Shenzhen Key Laboratory of Thermoelectric Materials, Department of Physics, Southern University of Science and Technology, Shenzhen 518055, China.

^9^Guangdong Provincial Key Laboratory of Advanced Thermoelectric Materials and Device Physics, Southern University of Science and Technology, Shenzhen 518055, China.

^10^Advanced Research Institute of Multidisciplinary Sciences, Qufu Normal University, Qufu 273165, China.

^11^Department of Physics and Astronomy, University of Missouri, Columbia, Missouri 65211, USA.

^12^Key Laboratory of Artificial Structures and Quantum Control, School of Physics and Astronomy, Shanghai Jiao Tong University, Shanghai 200240, China.

^13^State Key Laboratory of Structural Chemistry, Fujian Institute of Research on the Structure of Matter, Chinese Academy of Sciences, Fuzhou 350002, China.

†These authors (Peng-Fei Liu and Xiyang Li) contributed equally to this work.

*Corresponding authors: Hua Lin (linhua@fjirsm.ac.cn), (Junrong Zhang) jrzhang@ihep.ac.cn, Jiaqing He (hejq@sustech.edu.cn), Bao-Tian Wang (wangbt@ihep.ac.cn).


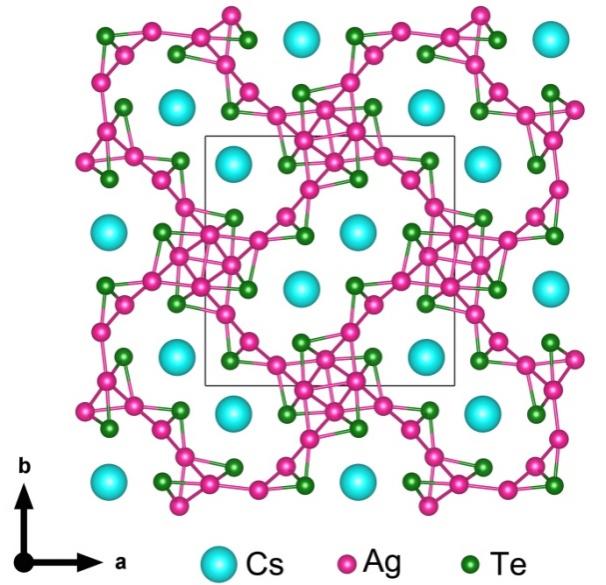


**Figure S1.** **Crystalline structure.** The top view of the crystalline structure of CsAg_5_Te_3_ with the two open Ag-Te tunnels filled up with Cs atoms. The unit cell is indicated by the solid black lines. The olive, green, and pink balls represent the Te, Cs, and Ag atoms, respectively.


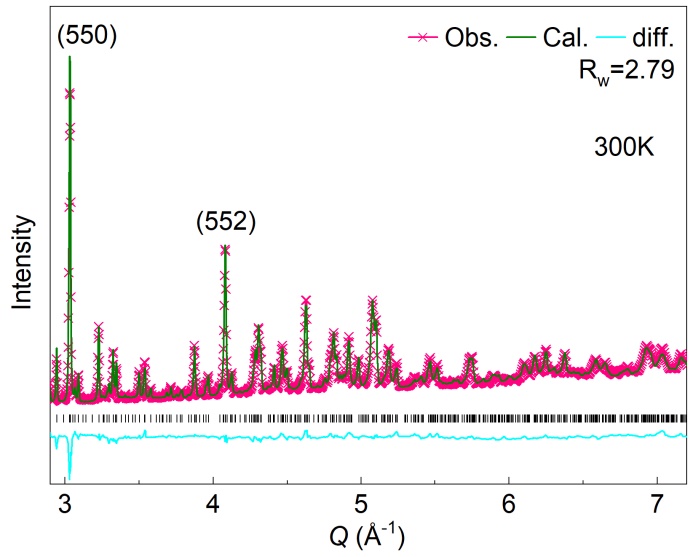


**Figure S2.** **Neutron diffraction.** Rietveld refinement of CsAg_5_Te_3_ neutron diffraction data measured at 300 K with two strongest peaks at (550) and (552).


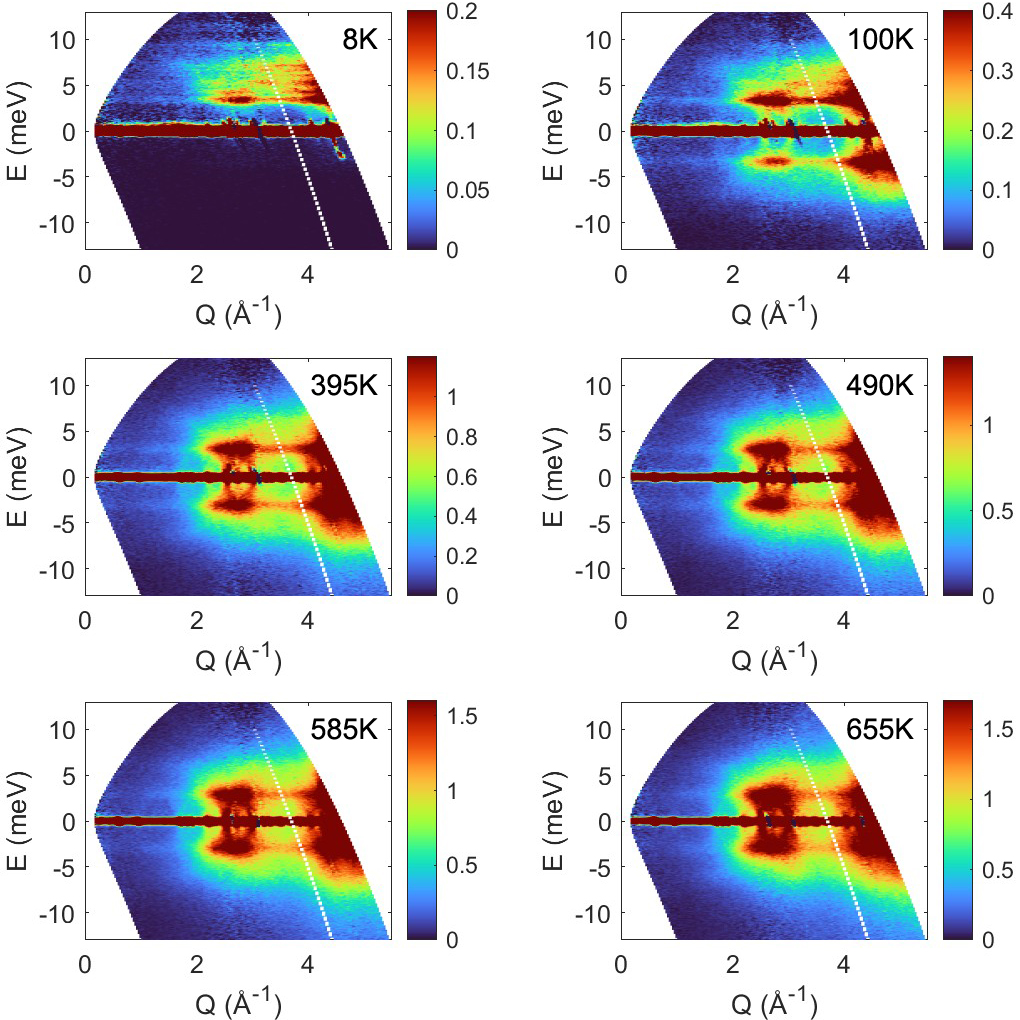


**Figure S3.** **INS data of phonons.** The contour plots of the dynamic structure factors *S*(**Q**,E) obtained by INS with an incident neutron energy of *E*_i_ = 15.15 meV from AMATERAS measurements at 8, 100, 395, 490, 585, and 655 K.


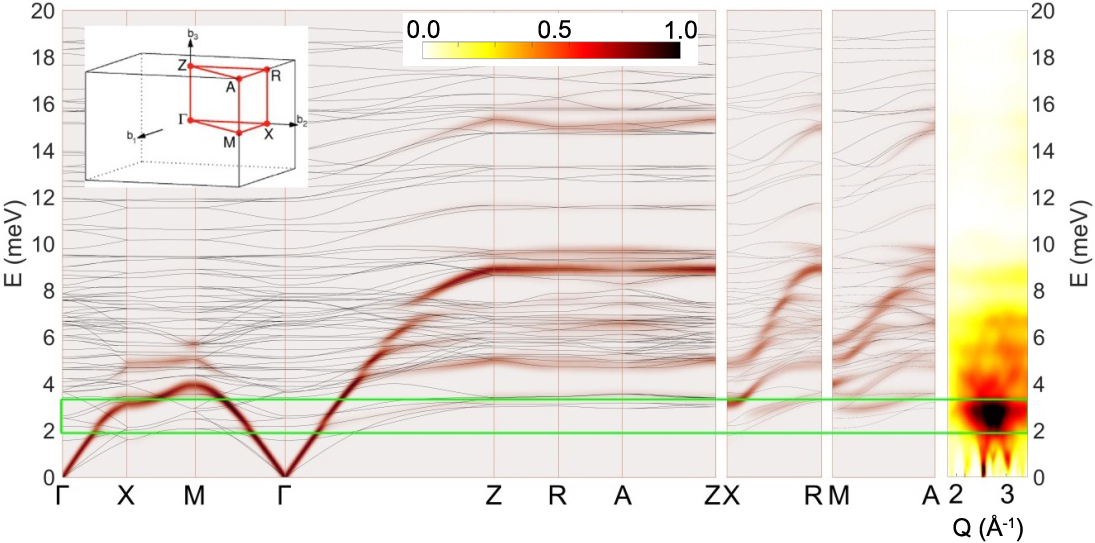


**Figure S4.** **Phonon dispersion.** Calculated phonon dispersion with the color being proportional to the magnitude of the neutron dynamical structure factor at 300 K. Right: The contour plot of the dynamic structure factor S(Q,E) ranging from 1.8 to 3.5 Å^-1^ at 300 K generated by the temperature-dependent force constants via first-principles calculations. Inset: Schematic of the 3D Brillouin zone with high-symmetry points marked by red points. The green rectangle in the low-energy region from 2.0 to 3.5 meV highlights the strong scattering pattern by INS. In this figure, we have normalized the S(Q,E).


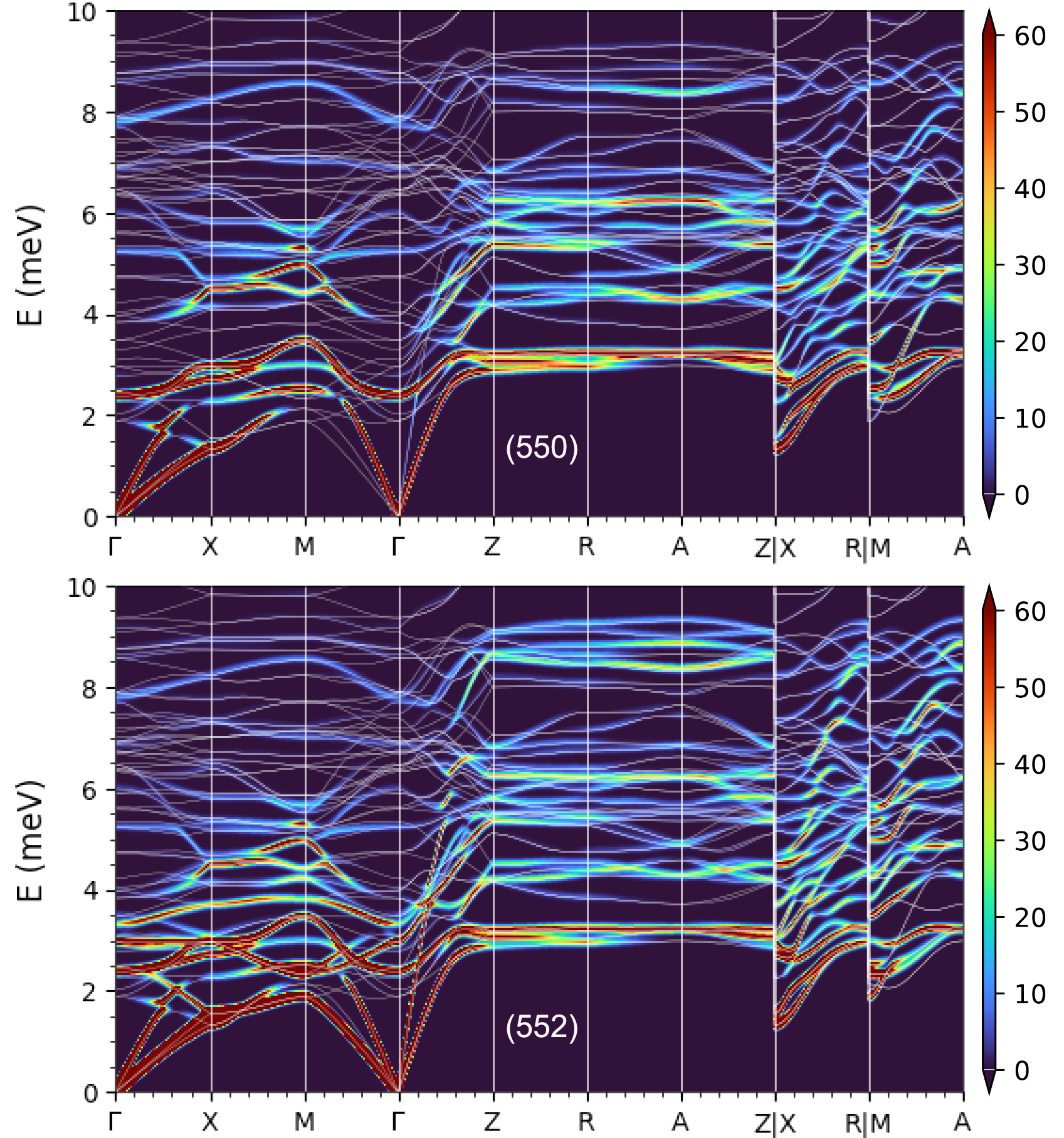


**Figure S5.** **Phonon dispersion.** Phonon dispersion with the color being proportional to the magnitude of the neutron dynamical structure factor at 300 K calculated with Euphonic for the Brillouin zones centered at the (550) and (552) zones. The color saturation increases linearly from zero to the calculated maximum value of S(Q,E). Those predicted phonon spectrums will undergo validation through experiments conducted on single crystals, notably employing techniques like INS. The visible TA modes are mainly due to the large Q at (550) and (552) zones, which are far beyond the first Brillouin zone.


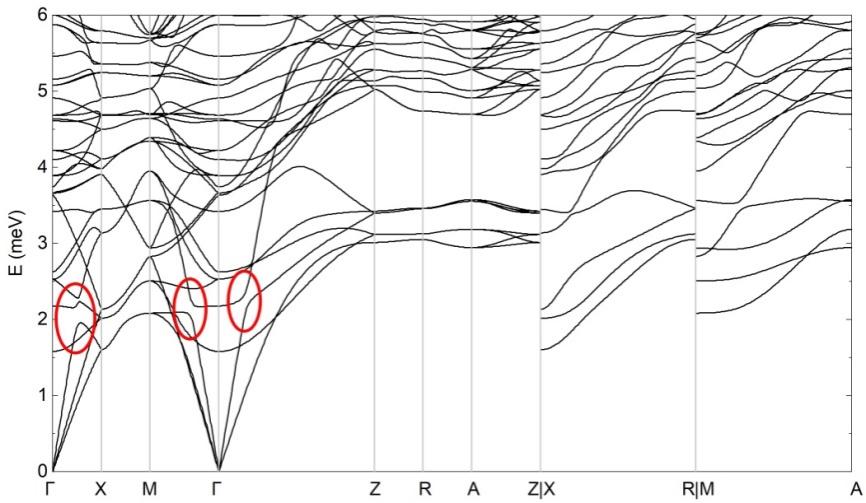


**Figure S6.** **Phonon dispersion.** Calculated phonon dispersion with the avoided-crossing feature of acoustic and optical phonon branches being marked by red circles.


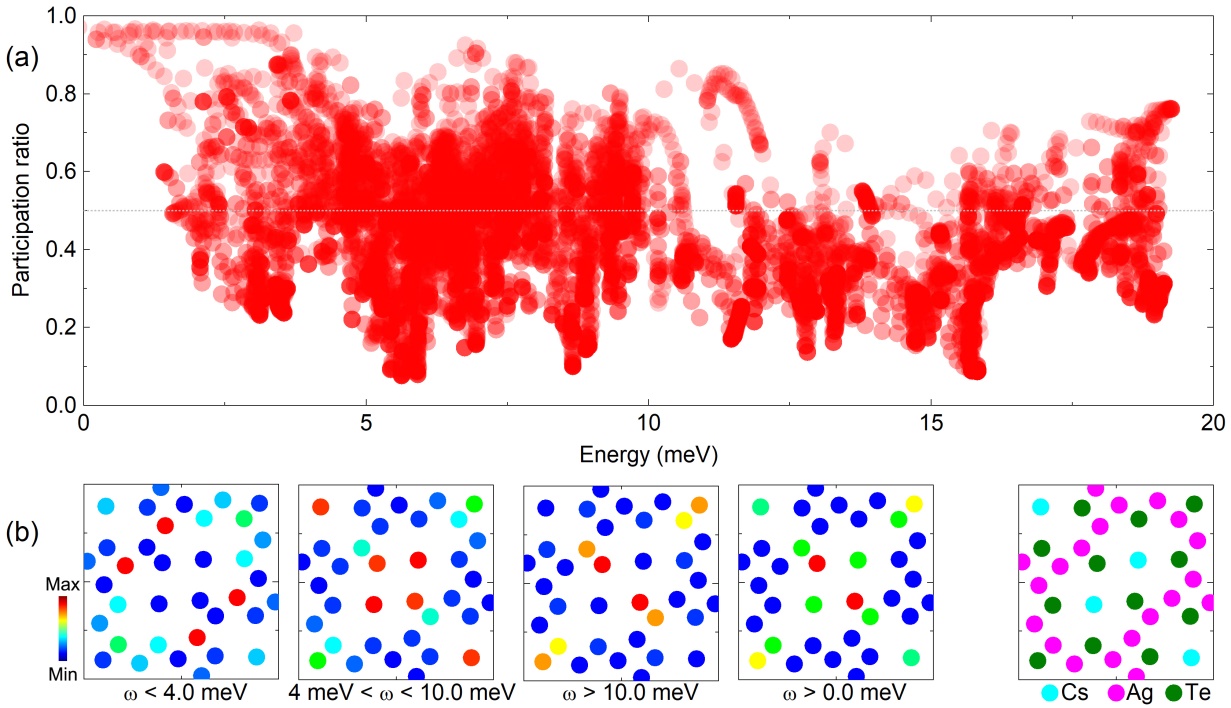


**Figure S7. Localized phonon modes.** (a) The calculated participation ratios for CsAg_5_Te_3_ obtained by the temperature-dependent force constant from MD at 300 K. It is calculated by $p\left( \omega_{\boldsymbol{q}} \right)=\frac{\left( \sum_{i=1}^{N} \left| \boldsymbol{u}_{i}\left( \omega_{\boldsymbol{q}} \right) \right|^{2} \right)^{2}}{N\sum_{i=1}^{N} \left| \boldsymbol{u}_{i}\left( \omega_{\boldsymbol{q}} \right) \right|^{4}}$, where $\boldsymbol{u}_{i}$ are the atomic amplitudes.The PR value of the propagative phonon mode, in which most of the atoms participate, is close to unity, while for a highly localized mode, the PR is on the order of O(1/N). Meanwhile, the PR values of the diffusive phonon modes are around ∼0.5, which is the character of the amorphous materials. (b) The four pictures on the left show the spatial distributions of the phonon modes in CsAg_5_Te_3_ with the different phonon frequency regions and *p*(ω_q_) < 0.2. It is calculated according to the formula $\phi_{i,\omega}=\frac{\sum_{\omega\in\Lambda} \sum_{\alpha} \varepsilon_{i\alpha,\omega}^{*}\varepsilon_{i\alpha,\omega}}{\sum_{j} \sum_{\omega\in\Lambda} \sum_{\alpha} \varepsilon_{i\alpha,\omega}^{*}\varepsilon_{i\alpha,\omega}}$, where α and i correspond to the Cartesian component (x, y, or z) and the atom index, respectively. Λ denotes a frequency range. The color bar indicates the portion of the local modes at a given frequency range. The rightmost subplot shows the atomic positions of Cs, Ag, and Te atoms.


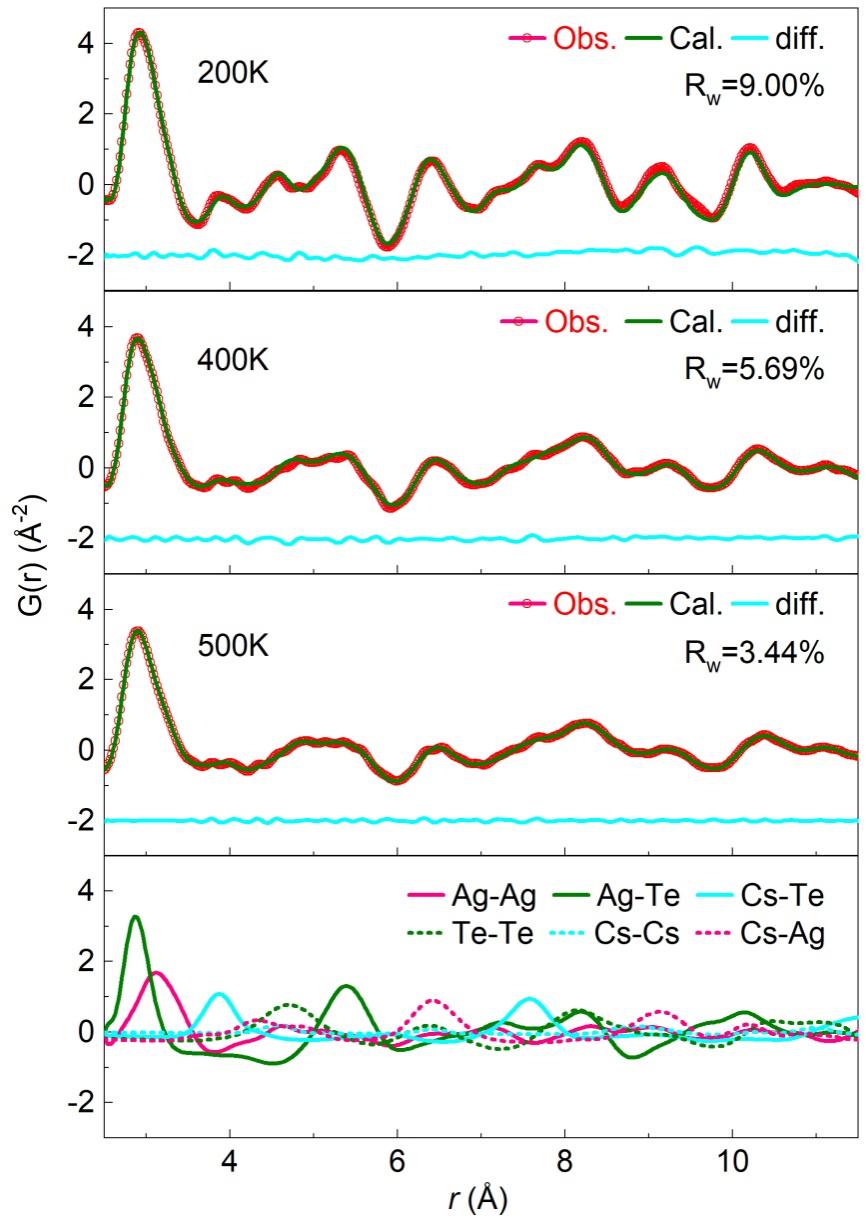


**Figure S8.** **Neutron PDF data.** Neutron PDF data is refined using *P*4_2_/*mnm* space group (ambient crystal structure) with all atom positions and thermal parameters refined at indicated temperatures. Partial PDF directly reveals there are no shoulder peaks from bonds between atoms.


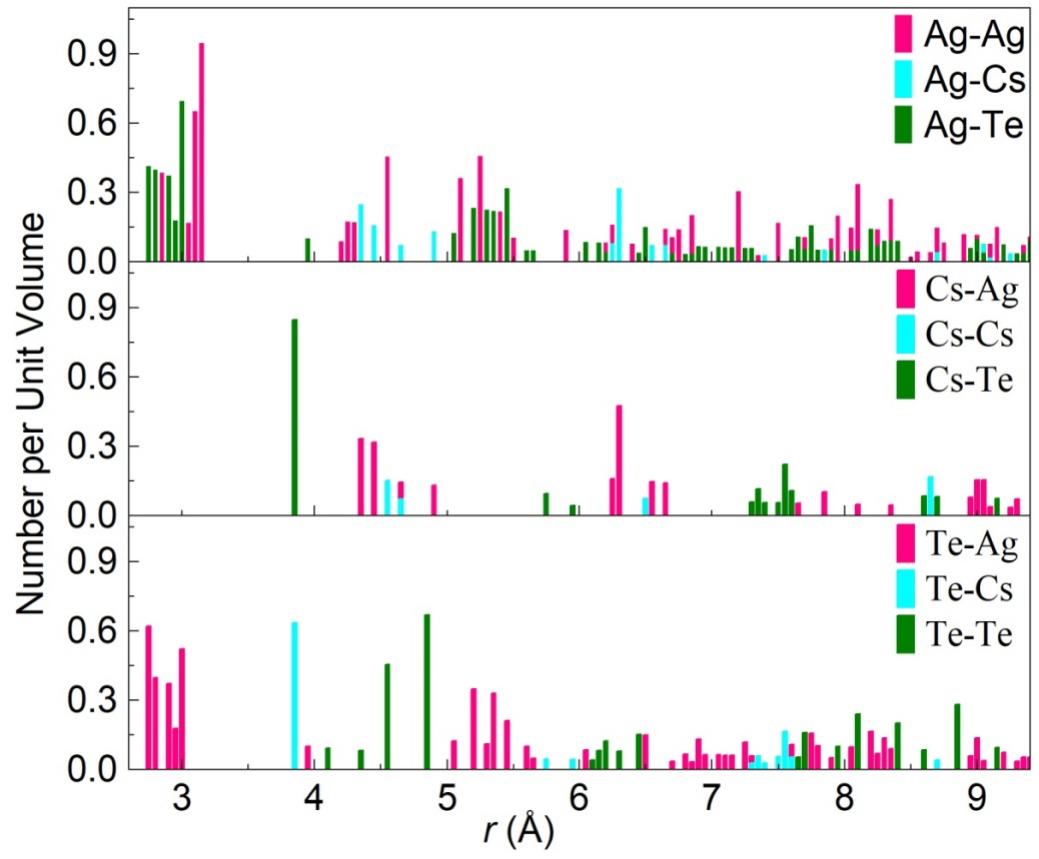


**Figure S9.** **Bond distances.** Bond-distance histogram from Ag, Cs, and Te to all atoms for any sites in the structure of CsAg_5_Te_3_. The olive, green, and pink columns represent the distances to Te, Cs, and Ag atoms, respectively.


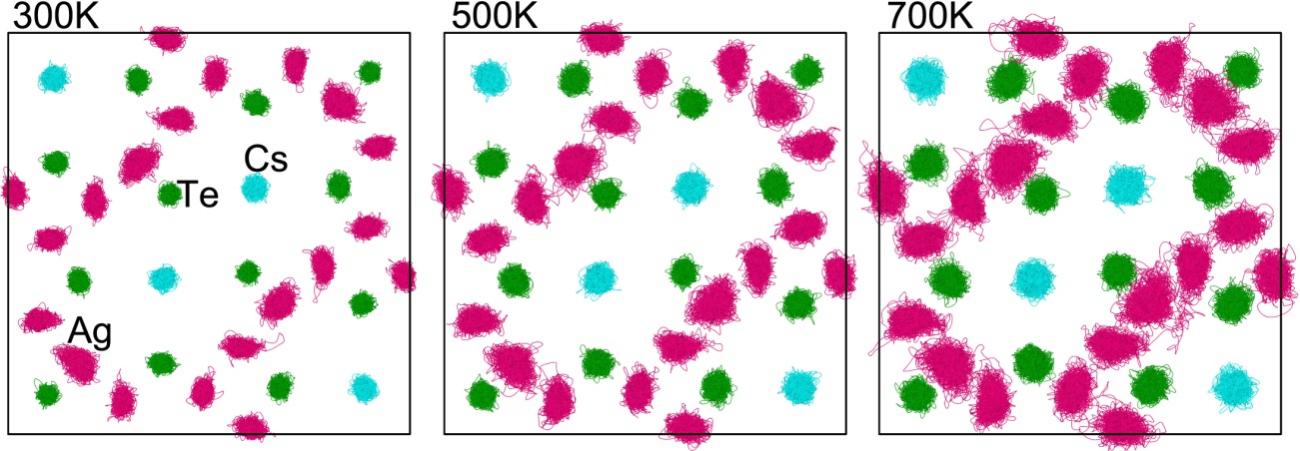


**Figure S10.** **Atomic trajectory.** Trajectory of atoms in the x-y plane from molecular dynamics simulations at 300, 500, and 700 K for CsAg_5_Te_3_. The olive, green, and pink balls represent Te, Cs, and Ag atoms, respectively.


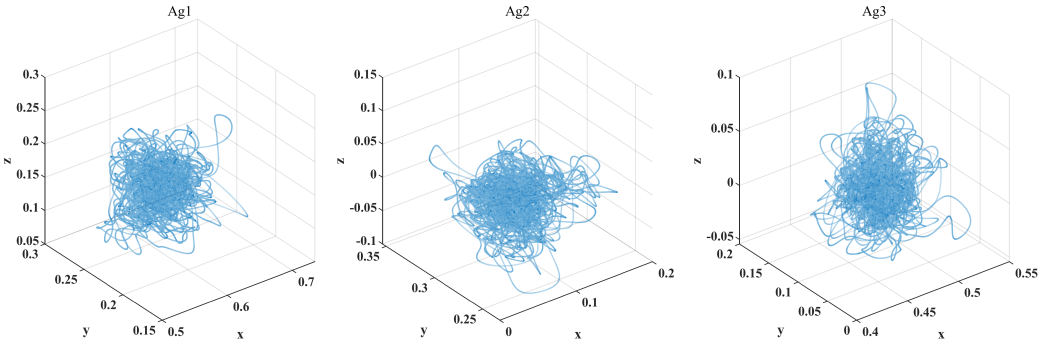


**Figure S11.** **Trajectory of Ag atoms.** Trajectory of the three Ag atoms in the 3D space from molecular dynamics simulations at 700 K for CsAg_5_Te_3_. It clearly shows that the Ag atoms are still constrained to around their equilibrium positions, despite having large oscillation amplitudes.


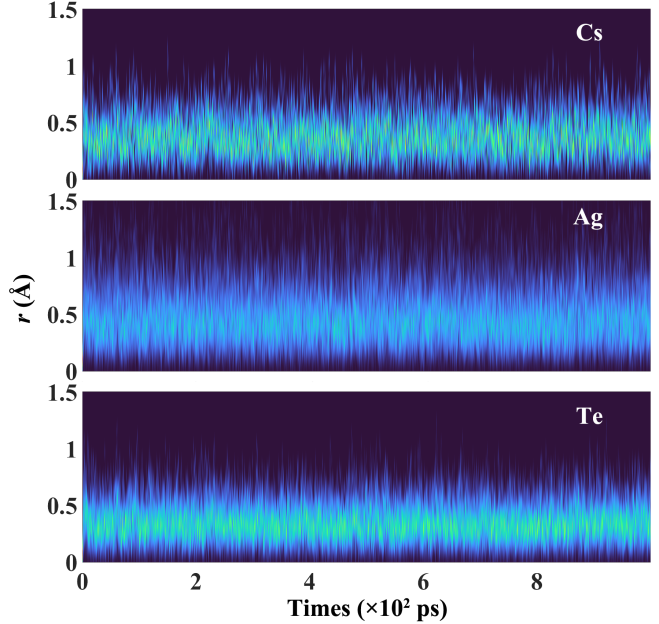


**Figure S12.** **Correlation functions.** Calculated self-part of van Hove correlation functions at 700 K for Cs, Ag, and Te atoms. The shining region shows the range of atomic fluctuations as a function of time. It depicts the probability that a particle will diffuse away from its initial positions by a distance r after a period t. As time passing, it fluctuates nearly around a fixed value for each atom. There is no jump diffusion for all atoms in CsAg_5_Te_3_. This is different with the superionic thermoelectric AgCrSe_2_, in which the quasi-2D liquid-like diffusion exists. This means that the Ag atoms in CsAg_5_Te_3_ have large oscillation amplitudes with fluid-like fluctuations but act as rattlers restricted to their equilibrium positions.


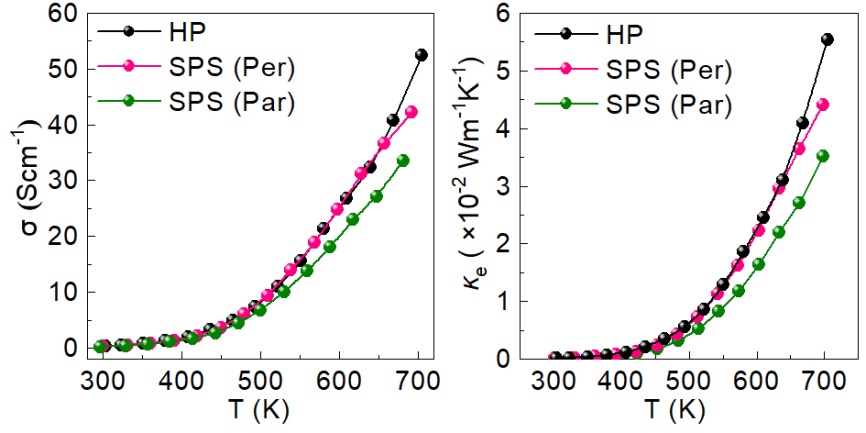


**Figure S13.** **Electrical transport.** Temperature dependence of electrical conductivity σ and electron thermal conductivity κ_e_ calculated by κ_e_ = LσT with the Lorenz number (L) being 1.5×10^−8^ V^2^K^−2^.


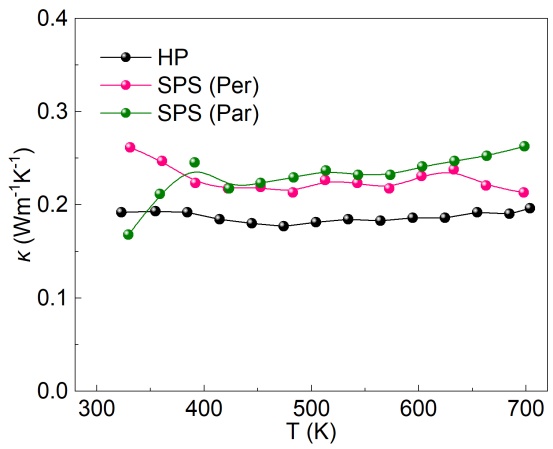


**Figure S14.** **Heat transport.** Temperature dependence of total thermal conductivity κ for CsAg_5_Te_3_. “Par” and “Per” mean the κ along parallel and perpendicular to the pressing directions with the SPS method. “HP” represents the κ of the hot-pressed sample.


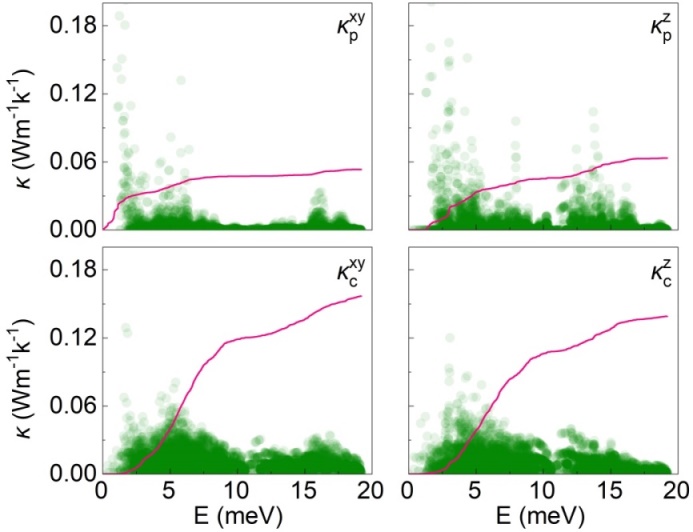


**Figure S15.** **Frequncy-dependence** **κ.** The frequency dependence of the modal (olive) and cumulative (pink) thermal conductivity from particlelike and wavelike phonon contributions along the in-plane and out-of-plane directions at 300 K.


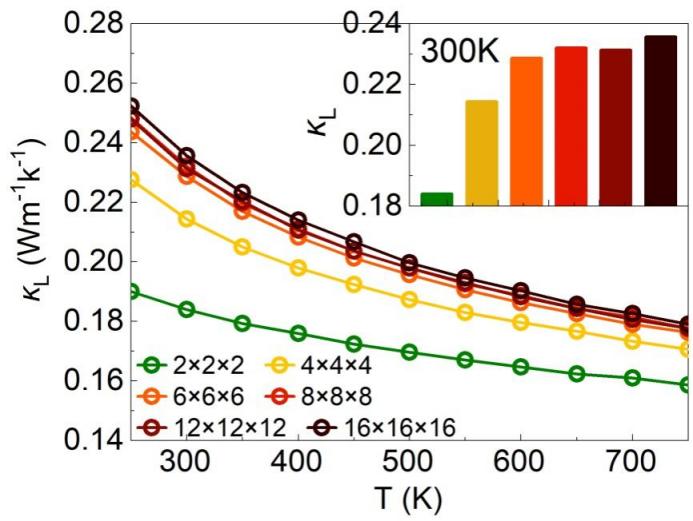


**Figure S16.** **Convergent κ_L_.** Temperature dependence of κ_L_ for CsAg_5_Te_3_ calculated with different numbers of **q**-point meshes along each axis. Insert: the κ_L_ at T = 300 K. In this test, the second-order force constants from MD at 300 K are used as input files.


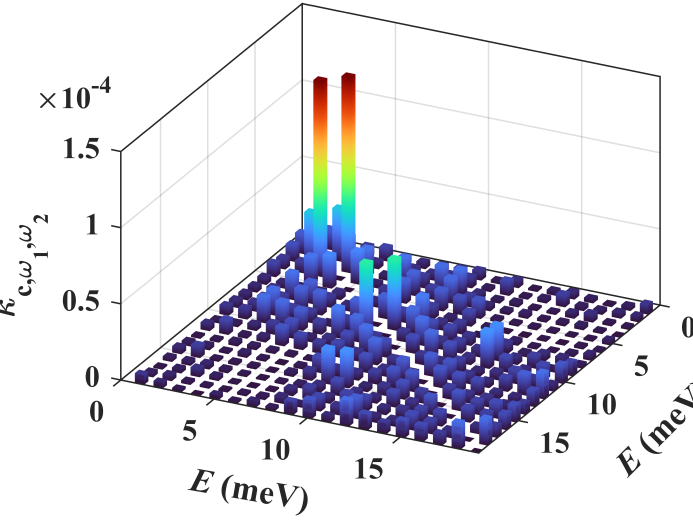


**Figure S17.** **Two-dimensional κ_c,ω1ω1_.** Two-dimensional density of states for the thermal conductivity, κ_c,ω1ω1_, which resolves how much a Zener-like coupling between two vibrational modes having frequencies ω1ω2 contributes to the coherence conductivity. Clearly, the major contributors to κ_c_ locate near the diagonal of the frequency plane with quasidegenerate vibrational frequencies as evidences for the existence of wavelike interband (Zener) tunneling of phonons.


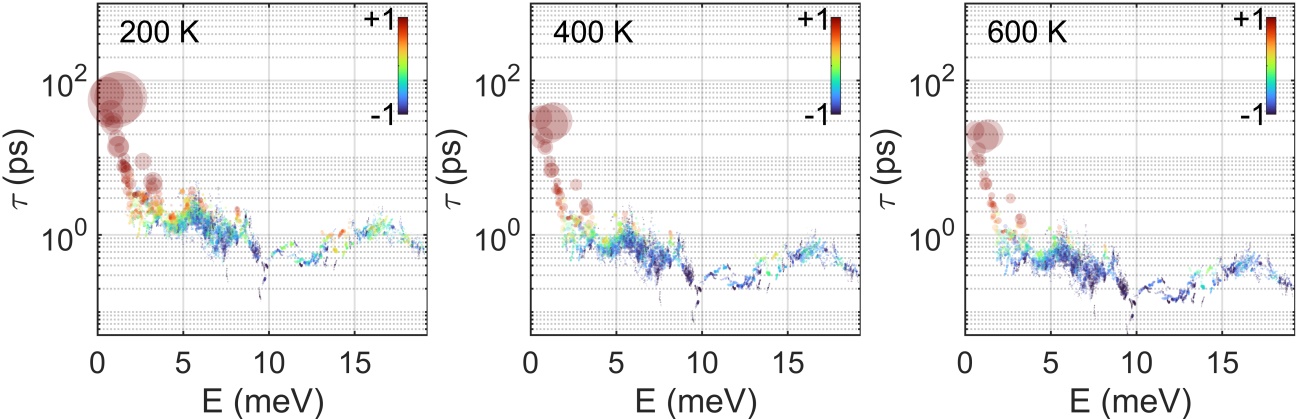


**Figure S18.** **Phonon lifetimes in CsAg_5_Te_3_.** Calculated phonon lifetimes τ(q) = [Γ(q)]^−1^ as a function of the energy ω(**q**) for CsAg_5_Te_3_ at 200, 400, and 600 K. The area of each circle is proportional to the contribution to the κ_L_ and the colors indicate the origin of the contribution by *c* = 1 for particle-like propagation of the populations, *c* = −1 for wave-like tunneling of coherences, and the intermediate values for phonons contributed from both mechanisms. Clearly, with temperature increasing, the proportion of coherences contribution increases.


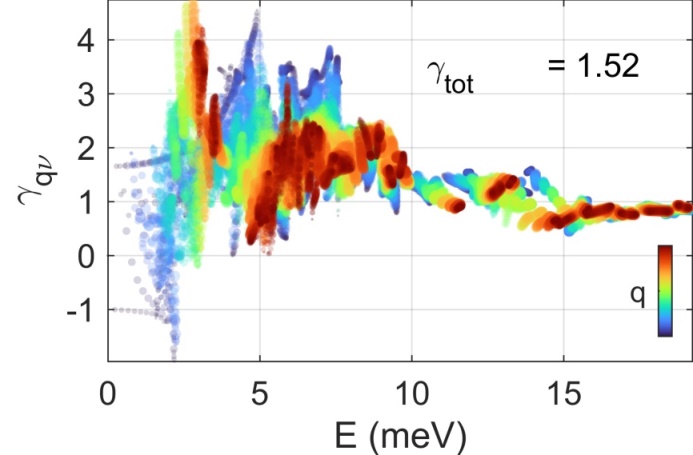


**Figure S19.** **Anharmonicity.** Calculated Grüneisen parameters γ_qν_ for CsAg_5_Te_3_. The color means the values at different **q** points. The inset shows γ_tot_ obtained as a weighted sum of the mode contributions for all phonons. The large values of γ**_q_**_ν_ are mainly accumulated in the range of 2∼10 meV, corresponding to the phonon modes that survive under INS.

**Table S1.** Calculated elastic constants (in GPa), Bulk Modulus *B* (in GPa), Young's Modulus *E* (in GPa), and Shear Modulus *G* (in GPa) for CsAg_5_Te_3_. Usually, soft bond strength always corresponds to low *E*, contributing to large lattice anharmonicity and low thermal conductivity. For CsAg_5_Te_3_, the Young’s modulus (E) is calculated to be 37.28 GPa. This value is lower than those of low-κ thermoelectric materials, such as PbTe (54.1 GPa) and α-MgAgSb (43.5 GPa), indicating the existence of much soft bonds in CsAg_5_Te_3_.

| *C*_11_ | *C*_12_ | *C*_13_ | *C*_33_ | *C*_44_ | *C*_66_ | *B* | *E* | *G* |
| --- | --- | --- | --- | --- | --- | --- | --- | --- |
| 56.84 | 19.51 | 17.48 | 63.45 | 11.88 | 10.05 | 31.77 | 37.28 | 14.29 |

**Table S2.** Calculated κ_L_, κ_p_, and κ_c_ (in Wm^−1^K^−1^) at 300 K for CsAg_5_Te_3_ with force constants being extracted by different methods.

| 2^nd^ IFCs | 3^rd^ IFCs | κ_p_ | κ_c_ | κ_L_ |
| --- | --- | --- | --- | --- |
| Phonopy | Phono3py | 0.09 | 0.14 | 0.23 |
| dynaphopy | Phono3py | 0.06 | 0.15 | 0.21 |
| TDEP | TDEP | 0.11 | 0.14 | 0.25 |
